# Revalidation and genetic characterization of new members of Group C (*Orthobunyavirus* genus, *Peribunyaviridae* family) isolated in the Americas

**DOI:** 10.1371/journal.pone.0197294

**Published:** 2018-05-24

**Authors:** Márcio Roberto Teixeira Nunes, William Marciel de Souza, Gustavo Olszanski Acrani, Jedson Ferreira Cardoso, Sandro Patroca da Silva, Soraya Jabur Badra, Luiz Tadeu Moraes Figueiredo, Pedro Fernando da Costa Vasconcelos

**Affiliations:** 1 Center for Technological Innovation, Evandro Chagas Institute, Ministry of Health, Ananindeua, Pará, Brazil; 2 Department of Pathology, University of Texas Medical Branch, Galveston, Texas, United States of America; 3 Virology Research Center, School of Medicine of Ribeirão Preto of University of São Paulo, Ribeirão Preto, São Paulo, Brazil; 4 Universidade Federal da Fronteira Sul, Campus Passo Fundo, Rio Grande do Sul, Brazil; 5 Department of Arbovirology and Hemorrhagic Fevers, Evandro Chagas Institute, Ministry of Health, Ananindeua, Pará, Brazil; Division of Clinical Research, UNITED STATES

## Abstract

Group C serogroup includes members of the *Orthobunyavirus* genus (family *Peribunyaviridae*) and comprises 15 arboviruses that can be associated with febrile illness in humans. Although previous studies described the genome characterization of Group C orthobunyavirus, there is a gap in genomic information about the other viruses in this group. Therefore, in this study, complete genomes of members of Group C serogroup were sequenced or re-sequenced and used for genetic characterization, as well as to understand their phylogenetic and evolutionary aspects. Thus, our study reported the genomes of three new members in Group C virus (Apeu strain BeAn848, Itaqui strain BeAn12797 and Nepuyo strain BeAn10709), as well as re-sequencing of original strains of five members: Caraparu (strain BeAn3994), Madrid (strain BT4075), Murucutu (strain BeAn974), Oriboca (strain BeAn17), and Marituba (strain BeAn15). These viruses presented a typical genomic organization related to members of the *Orthobunyavirus* genus. Interestingly, all viruses of this serogroup showed an open reading frame (ORF) that encodes the putative nonstructural NSs protein that precedes the nucleoprotein ORF, an unprecedented fact in Group C virus. Also, we confirmed the presence of natural reassortment events. This study expands the genomic information of Group C viruses, as well as revalidates the genomic organization of viruses that were previously reported.

## Introduction

Group C viruses are antigenically characterized into the genus *Orthobunyavirus*, family *Peribunyaviridae*, order *Bunyavirales* [1]. This name is historically based on their serological characteristics, which makes them distinct from members of group A (*Alphaviruses* genus of the family *Togaviridae*) and group B (*Flavivirus* genus of the family *Flaviviridae*) antigenic groups [2]. Currently, the group C serogroup is composed of 15 distinct viruses isolated from humans, wild animals (mainly rodents, monkeys, marsupials, and bats), and mosquitoes. These viruses are present in tropical and subtropical areas of the Americas, including the United States, Mexico, Panama, Honduras, Guatemala, Trinidad, Brazil, Peru, Ecuador, Venezuela, and French Guiana [1, 3, 4].

Clinically, the human infections caused by Group C viruses are asymptomatic or characterized by unspecific febrile illness [1, 5, 6]. The percentage of asymptomatic Group C viruses infections are unknown, and apparently, the prevalence of antibodies against Group C viruses is directly related to people living nearby or maintaining close contact to forest or ecological niches in tropical areas [5].

The genomes of members of Group C viruses presents the typical organization of other orthobunyaviruses, which are a tri-segmented negative-sense RNA named small (SRNA), medium (MRNA) and large (LRNA) segments. The SRNA encodes a nucleocapsid protein (N protein) and a non-structural protein (NSs), while the MRNA encodes a polyprotein precursor that after a post-cleavage process gives rise to two envelope glycoproteins (Gc and Gn) and a non-structural protein (NSm). LRNA segment encodes a large RNA-dependent RNA polymerase (RdRp) [7]. So far, previous studies have described the genomic characteristics S and M RNA segments of members of Group C viruses, but many sequences generated by Sanger sequencing approach were divergent, despite using the same strains of group C viruses [1, 7–9]. Therefore, in this study, we combined high-throughput sequencing (HTS), rapid amplification of cDNA ends (RACE), and comprehensive phylogenetic analysis of complete coding sequences of Apeu virus (APEUV) strain BeAn848, Itaqui virus (ITQV) strain BeAn12797, and Nepuyo virus (NEPV) strain BeAn10709 and re-sequencing of Caraparu virus (CARV) strain BeAn3994, Madrid virus (MADV) strain BT4075, Murucutu virus (MURV) strain BeAn974, Oriboca virus (ORIV) strain BeAn17, Marituba virus (MTBV) strain BeAn15.

## Material and methods

### Viruses and propagation in culture cells

Viruses strains used in this study were propagated into VERO cells (ATCC^®^ CCL-81^™^), as previously described [10]. The infected cells were incubated for 3 to 5 days until visualization of viral cytopathic effect. Table 1 provides the names, strains, year, sources and local of isolation, as well as the GenBank accession numbers.

**Table 1:** Group C viruses used in the present study.

Virus name	Abbreviation	Strain	Year of Isolation	Source of isolate	Location	Accession Numbers
*Apeu virus*	APEUV	BeAn848	10/14/1955	*Cebus apella*	Oriboca Forest, Pará State, Brazil	MG029269 to MG029271
*Itaqui virus*	ITQV	BeAn12797	09/02/1959	Sentinel mouse	Belém, Pará State, Brazil	MG029275 to MG029277
*Nepuyo virus*	NEPV	BeAn10709	1959	Sentinel mouse	Brazil	MG029287 to MG029289
*Caraparu virus*^*1*^	CARV	BeAn3994	02/15/1956	*Cebus apella*	Belém, Pará State, Brazil	MG029272 to MG029274
*Madrid virus*^*1*^	MADV	BT4075	03/03/1961	*Homo sapiens*	Almirante, Panama	MG029278 to MG029280
*Murucutu virus*^*1*^	MURV	BeAn974	1955	*Cebus apella*	Pará State, Brazil	MG029284 to MG029286
*Oriboca virus*^*1*^	ORIV	BeAn17	12/30/1954	*Cebus apella*	Oriboca Forest, Pará State, Brazi	MG029290 to MG029292
*Marituba virus*^*1*^	MTBV	BeAn15	12/27/1954	*Cebus apella*	Oriboca Forest, Pará State, Brazil	MG029281 to MG029283

Names, abbreviations, strain numbers, year, sources and locality of isolation and accession numbers of the viruses used in this study ^1.^ Viruses ressequencing.

### RNA extraction, construction of RNA library, sequence assembly and RACE assay

Viral RNAs were extracted from the supernatant of infected VERO cells using the PureLink Viral RNA Mini Kit (Invitrogen, USA) following the manufactures instructions. Then, the strands of the RNA synthesis were performed using the kit cDNA Synthesis System and 400 μM Roche “random” Primer according to the manufacturer’s instructions. The cDNAs were prepared for HTS on a GS FLX+ pyrosequencer (Roche, 454 Life Sciences) at the Center for Technological Innovation at the Evandro Chagas Institute, Ministry of Health, Brazil. The *de novo* assembling strategy applied to obtain the genomes was used with program Newbler v. 3.0 [11]. Additionally, sequences for terminal untranslated regions (UTRs) were determined by 5’/3’ rapid amplification of cDNA ends (RACE) sequencing (S1 Table) [12].

### Genomic characterization

Virus genomes were evaluated regarding it sizes, annotations of putative open reading frame (ORF), 5’ and 3’ non-coding regions (NCRs) and conserved motifs with Geneious 9.1.2 (Biomatters, New Zealand), for identification of transmembrane regions and signal peptide we used the TOPCONS web server [13] and for identification of N-glycosylation sites we used NetNglyc 1.0 Server (http://www.cbs.dtu.dk/services/NetNGlyc/). The annotations of protein domains were performed with InterPro 60.0 [14]. The presence of potential characteristic motifs for orthobunyaviruses were identified on multiple sequence alignments (MSA) based on amino acids sequences, which were carried out using Muscle v.3.7 [15], and visualized in Geneious 9.1.2 (Biomatters, New Zealand).

### Phylogenetic analysis and genetic distance

Maximum likelihood (ML) phylogenetic trees were constructed using nucleotide and amino acids sequences of viruses reported in our study and additional sequences of members of Group C viruses with complete coding sequences (S, M, and L) available in the GenBank database (http://www.ncbi.nlm.nih.gov/) until 10^th^ of April of 2018. The MSAs were carried out using Muscle v.3.7 [15]. The phylogenies were inferred by IQ-TREE version 1.4.3 software using the best-fit model based on Bayesian Information Criterion, and the GTR+I+G4 nucleotide substitution model was used to all RNA segments, and LG+G4, LG+I+G4, and LG+G4 amino acids substitution model to S, M, and L, respectively. Statistical supports for individual nodes were estimated using the bootstrap value using 1,000 replicates [16]. The phylogenetic trees were visualized using the FigTree software v.1.4.2. In addition, the nucleotides and amino acid distances among viruses of Group C were estimated with segment S, M, and L using the p-distance values. Standard error estimations were calculated by bootstrapping method (1,000 replicates) using the MEGA v.6 program [17].

### Reassortment events analysis

Potential reassortment events were analyzed by distinct phylogenetic topologies based on the depicted trees at the nucleotide level. In addition, all genes were concatenated in a single sequence, and an MSA was performed using the program Muscle 3.7 [15]. Potential reassortment events were then analyzed using the RDP, GENECONV, Bootscan, MaxChi, Chimaera, SiScan and 3Seq methods implemented in RDP4 [18]. Common program settings for all methods were used to perceive sequences as linear, to require phylogenetic evidence, to refine breakpoints and to check alignment consistency. The highest acceptable p-value was set as 0.05, after considering Bonferroni correction for multiple comparisons. All method-specific program settings remained at their default values.

## Results and discussion

### Genome organization of Group C viruses

**Fig 1:**
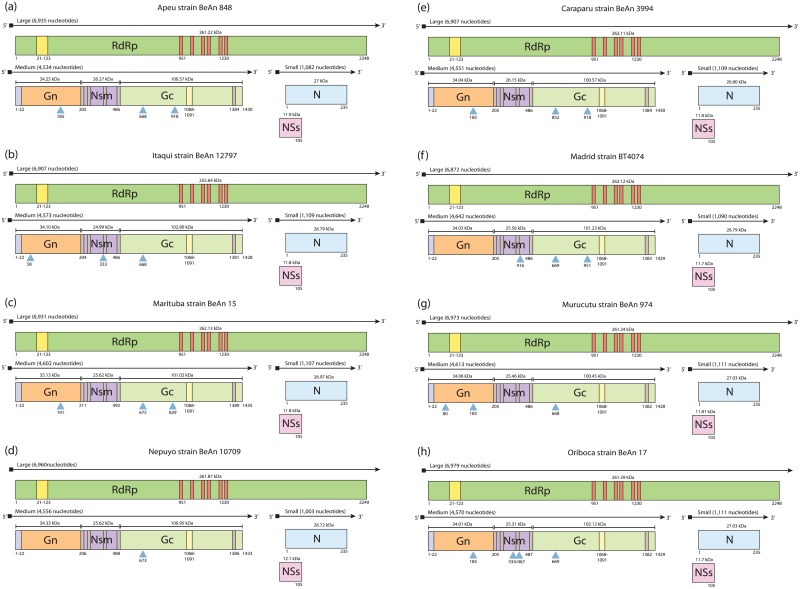
Genome organization of Group C viruses. (a) Apeu, (b) Caraparu, (c) Itaqui, (d) Madrid, (e) Marituba, (f) Murucutu, (g) Nepuyo and (h) Oriboca.

**Fig 2:**
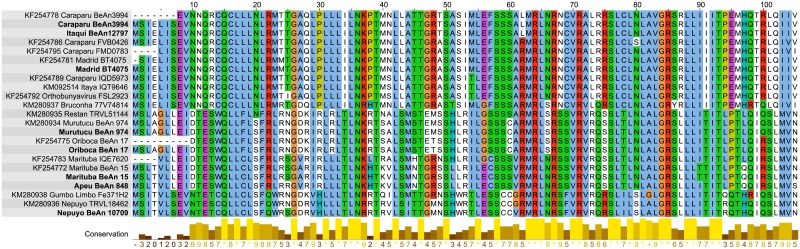
Alignment of non-structural protein of Group C viruses.

Our results showed that the complete SRNA segments of Group C viruses ranged from 1,003 to 1,111 nucleotides (nt) and presents the open reading frame (ORF) of the nucleocapsid (N) protein, with a conserved size of 235 amino acids (aa) and 26.72 to 27.03 kDa for all viruses (Fig 1). The second ORF in the S segment in these viruses encodes a putative non-structural protein (NSs). Interestingly, the ORF to NSs gene starts at an ATG codon -38 nucleotides downstream of the N ORF start codon, which potentially encodes an NSs protein of 318 nt (105 aa) with a molecular mass of ~11.8 kDa (Fig 1). The NSs protein is common in most orthobunyaviruses that have been isolated from vertebrates, such as the members of Group C viruses [19]. Previously studies have been reported that Caraparu and Madrid from Group C viruses encode an NSs protein with 83 amino acids, and also a 62 aa NSs, predicted to be truncated in the N-terminus of Marituba and Oriboca viruses, and consequently, could not be expressed [6, 7, 9]. Indeed, our analysis demonstrates that possibly the members of Group C viruses present a pre-N coding strategy, as recently described for the Brazoran and Enseada orthobunyaviruses [20, 21]. Therefore, some S segment sequences previously reported for Group C viruses are possibly incomplete, as shown in Fig 2. Whereas that NSs protein has been demonstrated to play crucial roles in virulence and pathogenesis of *Orhobunyaviruses* [19], further studies using reverse genetics may elucidate the biological importance of NSs protein found in viruses of Group C.

The complete MRNA segments ranged from 4,534 to 4,613 nt in length, which encodes the glycoprotein precursor (GPC), ranging from 1,428 to 1,435 aa in length (159.94 to 161.87 kDa) with a similar topology to other members of the *Orthobunyavirus* genus (Fig 1). The GPC is proteolytically cleaved to yield two structural glycoproteins, Gn (204 to 211 aa and 34.01 to 35.13 kDa) and Gc (942 to 947 aa and 100.45 to 102.8 kDa), and a nonstructural protein (NSm) with 281 or 282 nt (24.99 to 26.27 kDa) (Fig 1). The GPC contains the specific N-terminal signal peptide that is common to all members of the genus, which is responsible for delivering the nascent polypeptide to the endoplasmic reticulum [22]. The conserved Zinc finger region (amino acids position 257 to 295) and fusion peptide (amino acids position 1068 to 1091) in the GPC protein were also identified, which is an important RNA binding site and is probably involved in viral assembly [23]. Furthermore, the GPC was predicted to contain five transmembrane regions (TMDs): a single TMD in Gn (close to the C-terminus); three TMDs in NSm; and a single one, close to the C-terminus in Gc (Fig 1). The TMDs in Gn and Gc have been shown to play crucial roles in membrane fusion, assembly, and morphogenesis [24].

The complete LRNA segments ranged from 6,907 to 6,979 nt in length, with an ORF that encodes an RNA-dependent RNA polymerase (RdRp) of 2,248 aa, with a predicted molecular weight of 255.64 to 262.13 kDa. The unique exception is Nepuyo virus strain BeAn 10709 with a RdRp of 2,249 aa. This protein contains the conserved polymerase activity domains consisting of Pre-Motif A and Motifs A through E in position 951 to 1230 aa, as well as the N-terminal endonuclease motifs H, PD, and DxK (Fig 1). These domains are highly conserved in negative sense RNA viral polymerases as well as other members of the *Bunyavirales* order and are directly involved in the polymerase function activity [25]. In addition, we observed two mismatches in the 8th and 9th nucleotide of the S segments, and another mismatch in the 9th nucleotide of the L segments, but we did not found mismatches in the UTR of M segments (S2 Fig).

### Nucleotide, protein sequence conservation analysis and identification of variable regions

**Fig 3:**
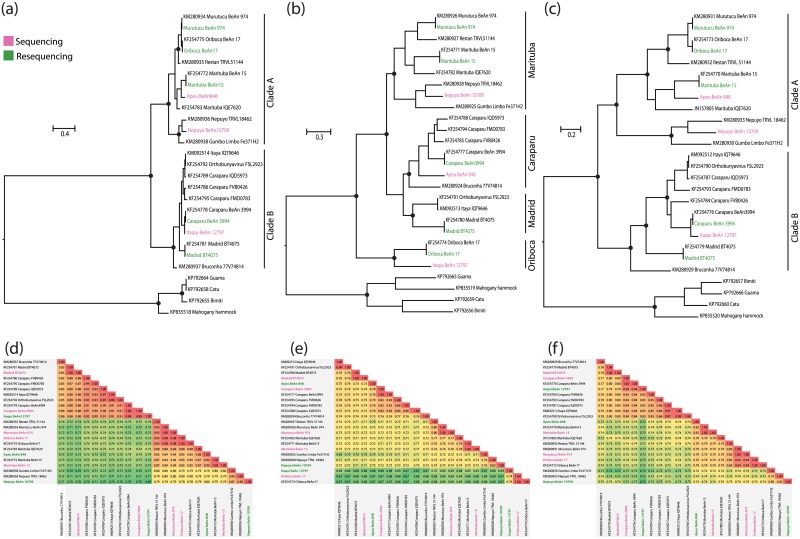
ML phylogenetic trees based on alignments of nucleotide sequences of Group C viruses. (a) S segment (b) M segment and (c) L segment. Phylogenies are midpoint rooted for clarity of presentation. The scale bar indicates evolutionary distance in numbers of substitutions per nucleotides substitutions/site, and the principal bootstrap support levels were indicated. Branches are colour-coded according to group. Viruses strains sequenced in this study are highlighted with red color. The Bimiti, Guama, Catu and Mahogany hammock were used as outgroup. Pairwise distance based on alignments of nucleotide sequences of Group C viruses with (d) S segment (e) M segment and (f) L segment.

MURV strain BeAn974, ORIV strain BeAn17, MTBV strain BeAn15, CARV strain BeAn3994 and MADV strain BT4075 viruses present 99.96 to 100% nucleotide identities with the corresponding partial genomic sequences that were previously reported (Fig 3D–3F **and Parts D-F of** S1 Fig) [4, 7, 8]. On the other hand, the new viruses that we have been sequenced the complete coding sequences shared higher nucleotide identify with partial genomes, such as Apeu strain BeAn 848, which shared 99% nucleotide identify in all segments with partial sequences of same strains previously described [9]. In addition, the amino acid substitutions were observed as follow: CARV strain BeAn3994 (S344P and K605N) on GPC. MTBV strain BeAn15 (Q335R); (L728Q); (Q1073K) and (K1345R) on RdRp and (G456R and D626N) in GPC. MURV strain BeAn974 (F949S) in RdRp. However, we did not observe any amino acid substitutions in the nucleoprotein sequence of the viruses that were submitted to the re-sequencing protocol. On the other hand, the identified amino acids substitutions observed in GPC and RdRp of our results compared with previously sequences reported to same strains. Therefore, we suspected that this fact is probably due to serial passages in cultured cells [26]. Collectively, our results are consistent with the same viral strains sequenced previously [4, 7–9], but the results of segment S suggested that some complete coding sequences previously reported for Group C viruses are possibly are incomplete.

### Evolutionary relationship of Group C viruses

To better understand the genetic relationships among group C viruses, we conducted the ML trees based on the complete coding sequences in nucleotide and amino acids level for all segments (Fig 3 and S1 Fig). These viruses were clustered in a unique monophyletic clade with different topologies for each segment. The S and L segments present two major clades, namely of clade A and B (Fig 3A–3C). The clade A was subdivided in three: subclade Ia, composed by MURV, ORIV, and Restan (RESV); subclade IIa, with MTBV and APEUV; subclade IIIa comprised by NEPV, Gumbo Limbo (GLV). The clade B was split into two subclades: subclade Ib, represented by CARV strains, ITQV, Itaya (ITYV) strain IQT9646 and FSL2923 and MADV and the subclade IIIb with Bruconha virus (BRUV) (Fig 3A–3C).

The topology of the ML tree for the M segment reveals four phylogenetic groups that were well supported (bootstrap > 80%). These groups were named following previous serological classifications based on complement fixation, neutralization, and hemagglutination inhibition tests: Marituba, Caraparu, Madrid and Oriboca complexes (Fig 3B) [1, 27]. The Caraparu complex includes CARV, APEUV and BRUV, Madrid complex comprises MADV and ITYV, Marituba complex comprises MTBV, MURV, RESV, NEPV and GLV, and the Oriboca complex was composed by ORIV and ITQV [1]. Unfortunately, there are no complete sequences available to Vinces and Ossa viruses, but probably these viruses will be clustered into Caraparu complex as previously showed by serological assays [28]. Possibly, the concordance observed between the phylogeny of the M segment and the serological classification occurs because the M segment encodes the glycoproteins, which are the primary antigenic determinants recognized by the neutralization and hemagglutination inhibition tests [1, 29].

### Reassortment events in Group C viruses

**Fig 4:**
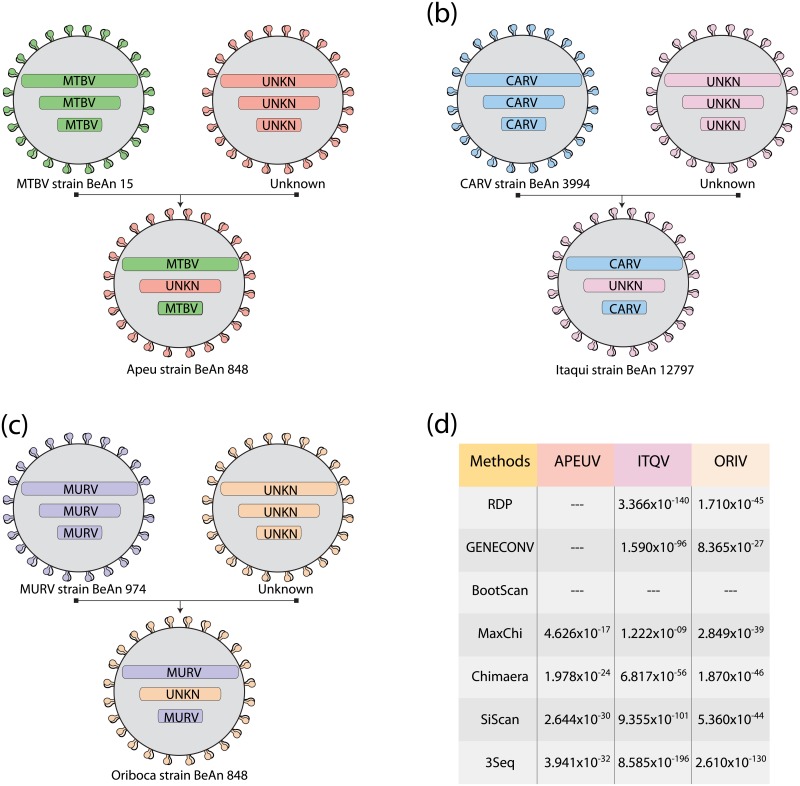
Reassortment events in Group C viruses. (a) Apeu, (b) Itaqui, (c) Oriboca and (d) Summary of RDP4 analysis to determine potential reassortants.

Reassortment is an important evolutionary mechanism of segmented RNA viruses in which co-infection of a host cell with multiple viruses may result in the shuffling of genomic segments [30]. This fact has been shown to be involved in virus emergence and interspecies transmission, which include Schmallenberg and Oropouche virus [31–33]. In Group C viruses, a classic and informative study using standard antigenic tests showed cross-reactivity in Marituba and Murutucu by hemagglutination-inhibition and neutralization, Murutucu and Oriboca by complement-fixation, Oriboca and Itaqui by hemagglutination-inhibition and neutralization, Itaqui and Caraparu by complement-fixation, Caraparu and Apeu by hemagglutination-inhibition and neutralization and Apeu and Marituba by complement-fixation [27]. However, some these viruses cross-reacted completely by one test, but not by another test and still are related. Thus, these and other data indicated that the Group C viruses include many natural reassortants [34]. To elucidate this point, we compared the topology of both S and L trees with the phylogenetic tree generated using the M segment sequences and observed some discrepancies, which were supported by higher bootstrap values and significantly different likelihood scores, which suggested natural reassortment. Also, after combining these branching inconsistencies in the phylogenetic trees with RDP4 analyses of concatenated segments, we confirmed four reassortment events with different genome segment organization than previously described [1, 7]. The first reassortment event was identified in APEUV strain BeAn848 contains S and L segments from MTBV strain BeAn15 and an M segment that is probably unique. Also, ITQV strain BeAn 12797 has S and L segments from CARV strain BeAn3994 and a possibly unique M segment. Interestingly, based on the classification of International Committee on Taxonomy of Viruses (ICTV), the reassortment events described in this study occurs between different viral species, as the Apeu virus that is classified into Caraparu orthobunyavirus, but the S and L this virus is from Marituba orthobunyavirus. Also, the Itaqui and Oriboca viruses are classified into Oriboca orthobunyavirus, but the S and L segments of both viruses are from viruses of Caraparu orthobunyavirus. Despite, previous studies based on serological tests and partial genome sequences indicated that APEUV and ITQV are potential reassortments, the first complete coding sequences for three segments were reported in this study [1, 7, 9, 34]. The other reassortment event was observed in ORIV strain BeAn17, which possesses the S and L segments from MURV strain BeAn974, and a unique M segment (Fig 4). Interestingly, all reassortments observed in this study have been reported cross-reactions only by the complement-fixation method [27]. This fact, suggests that cross-reactivity observed by complement- fixation test was determined by S segment because serologic makers identified by the L segment remains unidentified, as well as hemagglutination-inhibition, and neutralization assay that is determined by M segment, therefore, these viruses probably are unique [34]. Also, we confirmed the reassortments in ITYV identified in Amazon Region of Peru in 1999, both strains of this virus have S and L segments from Caraparu virus and a unique M segment [4]. On the other hand, MURV and RESV were previously reported to be reassortments, but we did not find any evidence to support this phenomenon [1]. Additional studies, as well as other strains of Group C viruses, may help to clarify this point.

In summary, our study provides a better understanding and clarifies the genome sequences, genomic characterization and reassortment events, as well as the evolutionary relationship of Group C serogroup. Thus, our results may be helpful to better understand the evolution and diversity of these viruses, as well as can be used in further molecular epidemiological, evolutionary studies, and development of molecular methods to diagnostic of infection by Group C viruses.

#### Nucleotide sequence accession number

The nucleotide sequence determined in this study have been deposited in GenBank under the accession number MG029269 to MG029292.

## Supporting information

S1 Fig ML phylogenetic trees based on alignments of amino acids sequences of Group C viruses. (a) Nucleoprotein (b) Glycoprotein precursor and (c) RNA-dependent RNA polymerase. Phylogenies are midpoint rooted for clarity of presentation. The scale bar indicates evolutionary distance in numbers of substitutions per nucleotides substitutions/site, and the principal bootstrap support levels were indicated. Branches are color-coded according to group. Viruses strains sequenced in this study are highlighted with red color. The Bimiti, Guama, Catu and Mahogany hammock were used as outgroup. Pairwise distance based on alignments of amino acids sequences of Group C viruses with (d) Nucleoprotein (e) Glycoprotein precursor and (f) RNA-dependent RNA polymerase.
(EPS)

S2 Fig Untranslated regions of Group C viruses obtained by RACE assay. (TIFF)

S1 Table Primers used to RACE assay. (XLSX)
