# Supplementary figures and images for: Revalidation and genetic characterization of new members of Group C (Orthobunyavirus genus, Peribunyaviridae family) isolated in the Americas
Source: PLoS One. 2018 May 24;13(5):e0197294. doi: 10.1371/journal.pone.0197294 (PMC5967719; doi:10.1371/journal.pone.0197294)

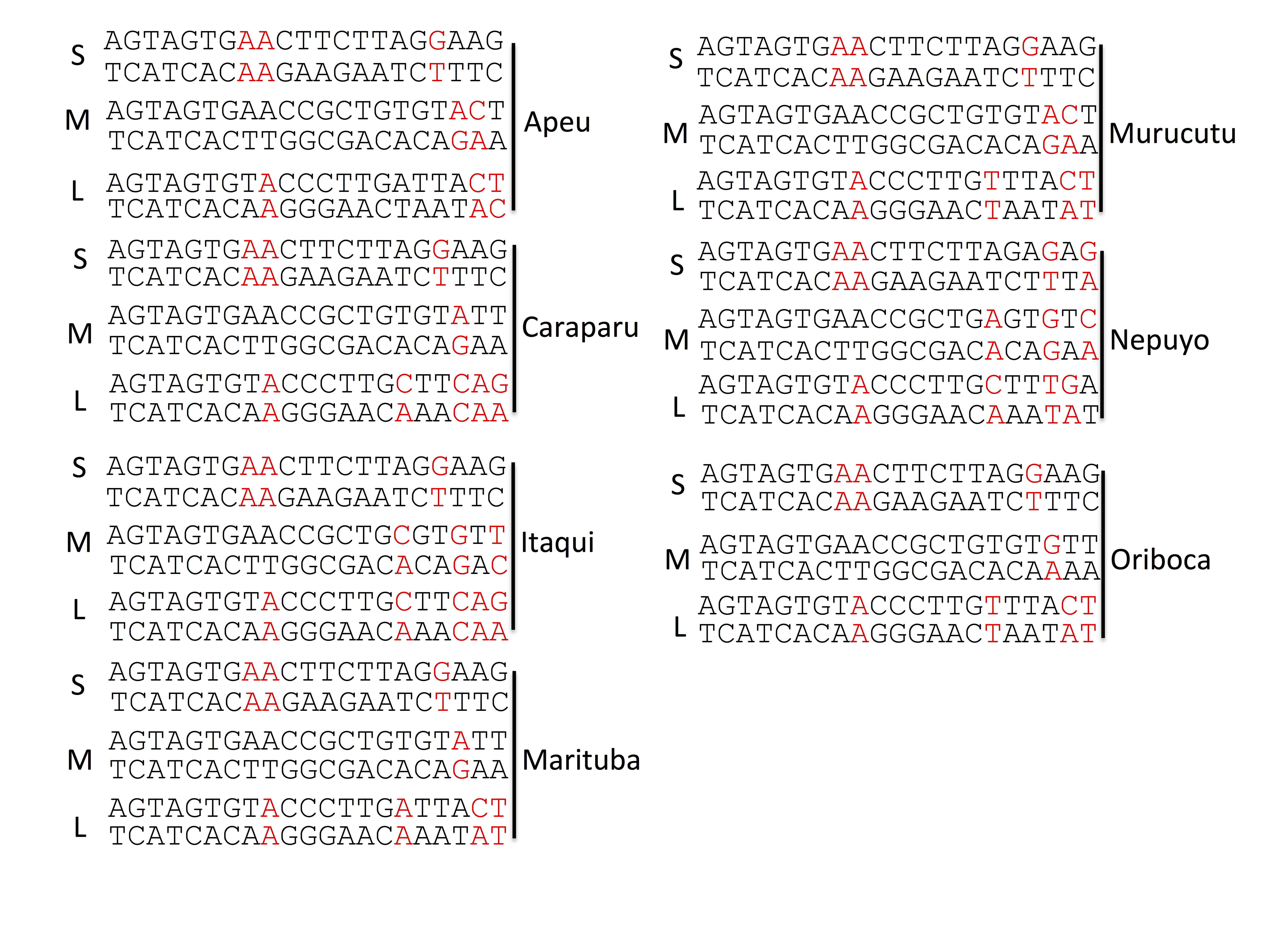

Supplement: S2 Fig — (TIFF) [file pone.0197294.s002.tiff]
